# Pharmacometabolomics of Bronchodilator Response in Asthma and the Role of Age-Metabolite Interactions

**DOI:** 10.3390/metabo9090179

**Published:** 2019-09-07

**Authors:** Rachel S. Kelly, Joanne E. Sordillo, Sharon M. Lutz, Lydiana Avila, Manuel Soto-Quiros, Juan C. Celedón, Michael J. McGeachie, Amber Dahlin, Kelan Tantisira, Mengna Huang, Clary B. Clish, Scott T. Weiss, Jessica Lasky-Su, Ann Chen Wu

**Affiliations:** 1 Channing Division of Network Medicine, Brigham and Women’s Hospital, Harvard Medical School, 181 Longwood Avenue, Boston, MA 02115, USA (R.S.K.) (M.J.M.) (A.D.) (K.T.) (M.H.) (S.T.W.) (J.L.-S.); 2 PRecisiOn Medicine Translational Research (PROMoTeR) Center, Department of Population Medicine, Harvard Medical School and Harvard Pilgrim Health Care, Boston, MA 02215, USA (J.E.S.) (S.M.L.); 3 Department of Pediatrics, Hospital Nacional de Niños, 10101 San José, Costa Rica (L.A.) (M.S.-Q.); 4 Division of Pediatric Pulmonary Medicine, UPMC Children’s Hospital of Pittsburgh, University of Pittsburgh, Pittsburgh, PA 15224, USA; 5 The Broad Institute, Cambridge, MA 02142, USA

**Keywords:** bronchodilator response, age by metabolite interaction, asthma, metabolomics, pharmacometabolomics, GABA, cholesterol esters, childhood asthma management program, genetic epidemiology of asthma in Costa Rica

## Abstract

The role of metabolism in modifying age-related differential responses to asthma medications is insufficiently understood. The objective of this study was to determine the role of the metabolome in modifying the effect of age on bronchodilator response (BDR) in individuals with asthma. We used longitudinal measures of BDR and plasma metabolomic profiling in 565 children with asthma from the Childhood Asthma Management Program (CAMP) to identify age by metabolite interactions on BDR. The mean ages at the three studied time-points across 16 years of follow-up in CAMP were 8.8, 12.8, and 16.8 years; the mean BDRs were 11%, 9% and 8%, respectively. Of 501 identified metabolites, 39 (7.8%) demonstrated a significant interaction with age on BDR (*p*-value < 0.05). We were able to validate two significant interactions in 320 children with asthma from the Genetics of Asthma in Costa Rica Study; 2-hydroxyglutarate, a compound involved in butanoate metabolism (interaction; CAMP: β = −0.004, *p = 1.8 × 10*^−4^; GACRS: β = −0.015, *p = 0.018*), and a cholesterol ester; CE C18:1 (CAMP: β = 0.005, *p = 0.006;* GACRS: β = 0.023, *p = 0.041*) Five additional metabolites had a *p*-value < 0.1 in GACRS, including Gammaminobutyric acid (GABA), C16:0 CE, C20:4 CE, C18.0 CE and ribothymidine. These findings suggest Cholesterol esters and GABA may modify the estimated effect of age on bronchodilator response.

## 1. Introduction

Asthma affects nearly 340 million people worldwide across all age groups and is responsible for roughly 1000 deaths every day [1]. Asthma arises from complex and insufficiently characterized nonlinear dynamic interactions between genes and environment. The pathogenesis of childhood-onset asthma differs from that of adult-onset asthma, and treatment response varies by age [2], suggesting that age plays a role in the underlying mechanisms of asthma phenotypes across the life-course.

Pharmaco-metabolomics is an emerging sub-discipline of metabolomics, which can be defined as the systematic analysis of the metabolites in a biological sample to improve understanding of the mechanistic effects of drugs, and the differences in response between individuals [3]. Metabolomics is particularly well suited to the study of asthma, as it reflects underlying genetics, environmental exposures, and phenotype. Consequently, pharmaco-metabolomics has the potential to inform precision medicine initiatives against asthma.

The Age-Dependent Pharmacogenomics of Asthma Treatment (ADAPT) study is a collaboration between multiple ongoing studies of asthma [4]. In this current study, we leverage two ADAPT cohorts of children with asthma: the Childhood Asthma Management Program (CAMP) and the Genetics of Asthma in Costa Rica Study (GACRS), to explore the role age plays in modulating the response to asthma medications utilizing metabolomics. The goal of this study was to determine the role of the blood metabolome in modifying the estimated effects of age on bronchodilator response (BDR) in individuals with asthma.

## 2. Results

### 2.1. Study Population

CAMP was a longitudinal cohort that recruited children with asthma and then followed them up for an average of 16 years. Over this time children returned for multiple in-person visits at which BDR was measured and blood was taken when possible. Blood samples were selected for metabolomics profiling in order to maximize the number of subjects with a sample at baseline, end of study and study follow-up. In total, 294 subjects with a sample at all three time-points; 265 subjects with a sample at two of the timepoints; and 6 subjects with a sample at only 1 time-point, were included in this analysis. The mean ages at the three time-points was 8.8 years. 12.8 years, and 16.8 years, respectively, and mean BDR (defined as the best forced expiratory volume in the 1st second (FEV_1_) post-bronchodilator minus FEV_1_ pre-bronchodilator, divided by FEV_1_ pre-bronchodilator, expressed as percentage) was 11%, 9% and 8%, respectively (Table 1). The study population was predominantly male (63–64% across the three time-points) and predominantly non-Hispanic white (70–72%). In GACRS, which was a cross sectional study with a single blood draw, 320 children were eligible for inclusion, with a mean age (9.1 years) and BDR (5.0%); comparable to the CAMP baseline; all participants were Hispanic (Table S1). 

### 2.2. Age*Metabolite Interactions

Of 501 named serum metabolites identified in CAMP, 39 (7.8%) demonstrated a significant interaction (*p* < 0.05) with age on BDR (Table 2) in a linear mixed model including race, sex, clinic, treatment group, age, metabolite, and the interaction term age*metabolite for the fixed effects. This model is designed to assess repeated measures from longitudinal data and can account for missing data enabling the inclusion of subjects who did not have samples for all three time-points of interest. The majority were lipids, specifically cholesterol esters, diacylglycerols and triacylglycerols; carnitine and bile acids. The top hit was 2-hydroxyglutarate, an alpha hydroxy acid form of glutaric acid (interaction beta: −0.004, 95% CI: −0.007, −0.002; *p-value = 1.8 × 10*^−4^*, q-value = 0.089*). There was evidence of high correlation between these metabolites; the cholesterol esters were highly correlated with each other at all three time-points, as were the tri-/di-acylglycerols, but cholesterol esters metabolites and the tri-/di-acylglycerol metabolites were inversely correlated with each other (Figures S1–S3). 2-hydroxyglutarate, sebacate, suberate and pimelate, which are all dicarboxylic acids, were highly correlated with each other across the three time-points. 

In GACRS (age range: 6 to 13 years), 12 (2.0%) of 615 metabolites had a significant interaction with age on BDR (*p*-value < 0.05) (Table S2) in a multivariable linear regression including sex, age at sample, metabolite, and the interaction term age*metabolite. As in CAMP, these were primarily lipids, including five cholesterol esters. The age*metabolite interactions on BDR in CAMP replicated in GACRS for 2-hydroxyglutarate (*p* = 0.018) and C18:1 CE (*p* = 0.041), with a consistent direction of effect. Five additional metabolites (C16:0 CE (*p* = 0.056), C20:4 CE (*p* = 0.076), GABA (Gamma-Aminobutyric acid) (*p* = 0.085), ribothymidine (*p* = 0.088) and C18:0 CE (*p* = 0.101)) had a *p*-value in GACRS <0.1 with a consistent direction of effect (Table 3).

In CAMP, after adjustment for race, gender, study center and treatment group, levels of 2-hydroxyglutarate increased with age, while levels of the cholesterol esters, GABA and ribothymidine decreased with age at the study end and follow-up (Table 4). There was no significant association at the baseline time-point, when the children were aged 5–13 years. Similarly, no significant associations between age and metabolite levels were observed in the GACRS participants, who were aged 6–13 years, after adjustment for gender.

**Figure 1:**
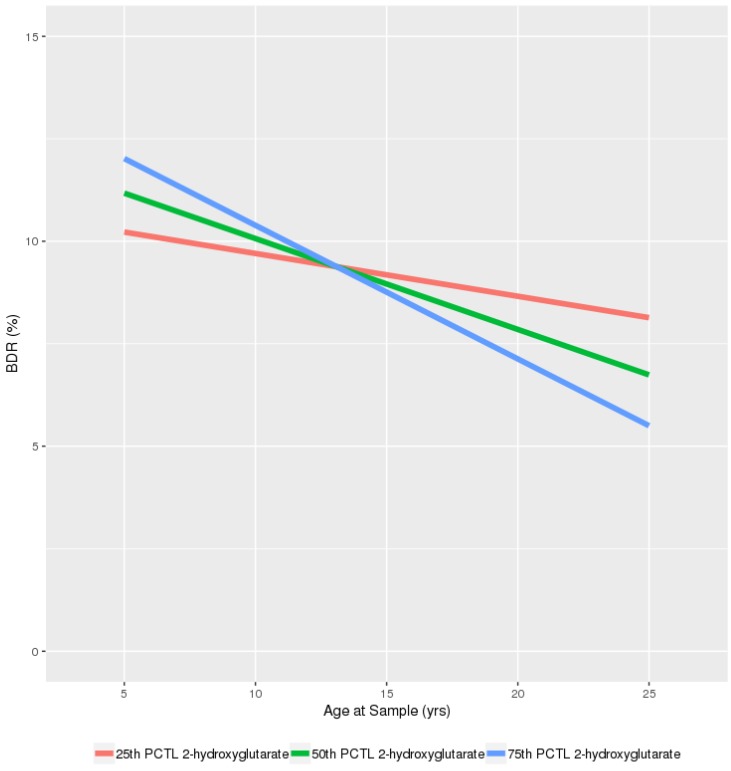
Predicted relationship between Age and BDR stratified by plasma levels of 2-hydroxyglutarate in CAMP (25th, 50th and 75th percentile). PCTL-Percentile; GABA—*Gamma-Aminobutyric acid;* *CE—Cholesterol Ester*.

**Figure 2:**
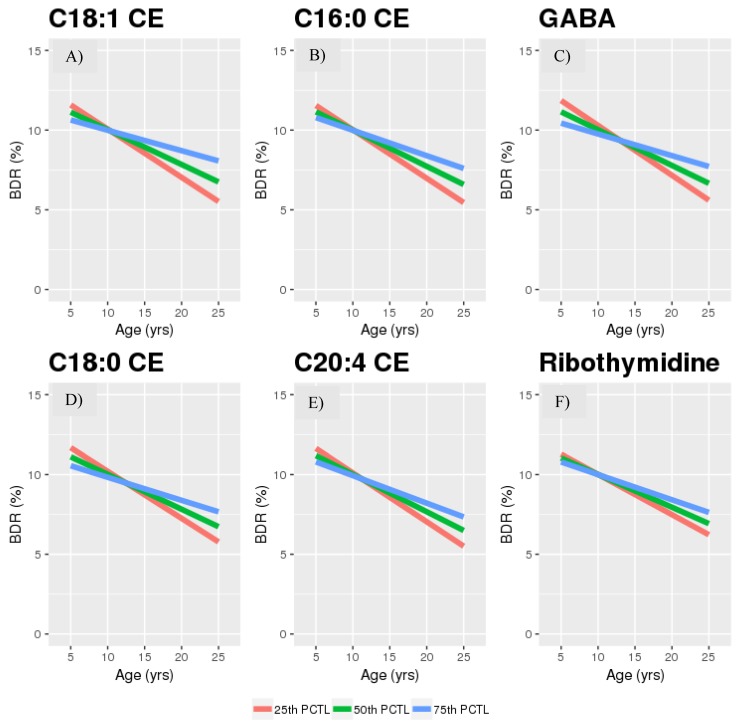
(**A**) C18:1 CE; (**B**) C16:0 CE; (**C**) GABA; (**D**) C18:0 CE; (**E**) C20:4 CE; (**F**) Ribothymidine in CAMP (25th, 50th, and 75th percentiles). Abbreviations: PCTL—percentile; CE—cholesterol ester GABA—gamma-aminobutyric acid.

The results suggest that there is an inverse association between BDR and age that intensifies with increasing levels of 2-hydroxyglutarate (Figure 1); those with higher levels of 2-hydroxyglutarate appear to experience greater age-related reductions in BDR. The opposite was true for GABA, ribothymidine and three cholesterol esters: the inverse association between BDR and age was somewhat attenuated with increasing levels of these metabolites. (Figure 2). In GACRS, due to the narrower range of ages, we only plotted between the ages of 5 and 15 years, however these plots did support the effect of these metabolites on the age-BDR relationship (Figures S4 and S5).

### 2.3. Sensitivity Analyses

Sensitivity analyses were performed to explore the role of race and gender. Genotyping was available for 495/560 of the subjects with samples at baseline; 500/563 at study endpoint and 263/295 at follow up; and were used to compute ancestry scores. When these were included in the model as the first four principal components, in place of race the results were largely unchanged (Table S3). All but three metabolites retained significance at *p* < 0.05, including the metabolites that we determined to be replicated in GACRS. We note that the GACRS population represents a semi population isolate, and subjects were selected on the basis of their genetic similarity, therefore although we have genotype data for these individuals, we do not adjust for ancestry score in this population.

We additionally ran these analyses stratifying by race in CAMP, although we were somewhat limited by sample size particularly for the Black and Hispanic participants; (White n = 1007 samples from 400 subjects; Black n = 198 samples from 82 subjects; Hispanic n = 133 samples from 56 subjects). We observed that 28 of the 39 metabolites of interest from CAMP were significant at a *p* < 0.05 in the White population; including 2-hydroxyglutarate (β = −0.003, *p = 0.018*); GABA (β = 0.003, *p = 0.014*), C16:0 CE (β = 0.006, *p = 0.009*); C20:4 CE (β = 0.004, *p = 0.030*), C18.0 CE (β = 0.003, *p = 0.063*); and ribothymidine (β = −0.003, *p = 0.018*) (Table S4). Of these only 2-hydroxyglutarate was significant in the Black participants (β = −0.011, *p = 0.010*); however the directions of effect were largely consistent across the races; and we postulate the lack of significant findings is likely to be driven by the limited sample size.

The sex-stratified results for CAMP (Table S5) and the GACRS (Table S6), seemed to indicate that the interactions with age may differ between males and females for some metabolites. For 2-hydroxyglutarate, similar age interaction patterns were seen in both sexes in CAMP (males β = −0.005, *p* = 0.002; females β = −0.004, *p* = 0.053), with some evidence that age related decreases in BDR by metabolite level may occur slightly earlier for females than for males (Figure S6). In the GACRS, a significant interaction was only found in males (β = −0.021, *p* = 0.015), but sample size was limited for females. For the cholesterol esters, significant interaction effects were largely observed in males only in both CAMP and the GACRS.

## 3. Discussion

Our results suggest that an inverse association between age and BDR in asthmatics aged 5–25 years may be enhanced with higher levels of 2-hydroxyglutarate, while increased levels of cholesterol esters, GABA and ribothymidine may attenuate the age-associated BDR decline. BDR, the change in airway constriction before and after the administration of a short-acting β 2 -agonist, is strongly correlated with asthma control [5], and has been shown to decrease with age [6,7]. Although most studies demonstrating age-related effects have been conducted in middle-aged individuals, there is some evidence to suggest decline may begin as early as adolescence [4,8].

2-hydroxyglutarate is an alpha-hydroxy acid form of glutaric acid. Although 2-hydroxyglutarate has not previously been associated with lung function or asthma, it has been associated with hypoxia in primary cultures of lung cells [9]. 2-hydroxyglutarate is involved in the butanoate metabolism pathway, which regulates the GAD (Glutamic Acid Decarboxylase)-mediated decarboxylation of L-glutamate into GABA. GABA, an inhibitory neurotransmitter, is known to participate in regulation of contractility of airway smooth muscle [10]. GABA has a number of positive effects on lung function, including relaxing airway smooth muscle, inhibiting muscle conduction, decreasing resistance in airway breathing channels, decreasing contractility of trachea muscle, reducing neurogenic extravasation, reducing anticholinergic bronchoconstriction, and regulating mucus hypersecretion [10,11]. Thus, increased levels of GABA metabolites may help to mitigate the decreases in BDR shown to accompanying aging. Further work is required to understand the role of 2-hydroxybutarate in this relationship.

Four highly correlated cholesterol esters were also among the replicated metabolites, based on a *p*-value of <0.1. Cholesterol esters are dietary lipids, which play an important role in the mediation of inflammation and immune function [12,13]. A dysregulated immune system is one of the key facets of asthma and asthmatic lung function, and immune function is known to decline with age [14]. Increased levels of some cholesterol esters may thus lessen age-associated immune and lung function changes.

In CAMP, GABA and the cholesterol esters were shown to decline with age, but this only reached significance amongst the older participants. Similarly, most associations were inverse but did not reach significance in the GACRS. There is scant literature on the association between age and blood GABA levels, but plasma levels of total cholesterol and cholesterol esters have been reported to increase with age in some studies [15], in contrast to our findings. However, these metabolites are highly influenced by diet, which is itself influenced by age, among multiple other factors. Our findings suggest a possible interaction between age and GABA and cholesterol esters, and individuals with high levels of these metabolites may be able to reduce the normal process of age-associated BDR decline. In particular, GABA and the GABAergic system have previously been proposed as a compelling new therapeutic avenue for asthma [11]. These findings suggest GABA may be particularly useful to help slow or reverse age-related decline in lung function.

Given that sex differences in asthma phenotypes may emerge in the window from childhood to adulthood, we examined the replicated age*metabolite interactions in sex-stratified models. There was some evidence for sex-specific variation, which may relate to observed differences in the metabolome that accompany puberty [16]. Intriguingly, the interaction of 2-hydroxyglutarate with age appeared to be shifted towards an earlier age in females, mirroring the age of pubertal onset which typically occurs earlier in females. The differences by sex in the cholesterol esters are also of note, given their observed associations with sex in the literature [17]. However, these stratified analyses were limited by small numbers and further studies are needed to determine whether age-related changes in metabolite profiles influence sex-specific lung function and asthma treatment responses.

One of the key strengths of this study is that we were able to identify metabolites that may help to understand the biology of age-related differences in therapeutic response in asthma. The key to precision medicine is a complete understanding of disease mechanisms; how these differ between individuals with the same apparent disease phenotypes and influence therapeutic response. Age has consistently been shown to be one of the key factors underlying these pathogenic and therapeutic differences among individuals with asthma. Although the number of metabolomic studies of asthma is increasing, most asthma studies are in either distinct adult or child populations, and studies in adolescent populations or that consider the influence of ageing are lacking [18]. Furthermore, few have considered the metabolomics of BDR or treatment response. This current study is unique in its utilization of metabolomic profiling to explore BDR among a population of asthmatics over multiple time-points spanning both childhood, adolescence, and early adulthood. An additional strength of this study was the ability to replicate our findings in an independent cohort.

Despite these strengths, a few caveats deserve mention. First, there were some notable differences between the studies, which may explain the limited replication. CAMP is a longitudinal study which encompassed a much wider age range than GACRS, which is cross-sectional in design with only a single time-point. This difference in age range may be of particular importance, as the greatest age related differences in metabolite levels in CAMP were shown to occur in late adolescence/early adulthood, an age range not captured by the GACRS population. The longitudinal nature of CAMP may also introduce bias as all samples from the three time-points were processed and sent for metabolomics profiling at the same time, meaning that the storage time that the samples experienced differed by as many as 16 years. It has been shown that increased storage time, as well as repeated freeze thaw samples can influence the quality and concentration of metabolites [19,20]. However, we note our samples were stored at -80C following best practice guidelines, and that this effect is metabolite class dependent and our metabolites of interest are not among those shown to be most affected [19].

Second, there were differences in the racial structure of the two cohorts; CAMP included multiple races, while all participants in GACRS were Hispanic. We adjusted for race in CAMP and we found that these results were largely unchanged when we additionally explored adjustment by ancestry score. however we were underpowered to stratify by race and therefore our race stratified results were largely non-significant, particularly among the Black and Hispanic populations. Nevertheless, we note that the directions of effects were largely consistent across the populations. We also have abundant data demonstrating that the asthma-relevant genetic findings in CAMP are generalizable to GACRS [21,22,23,24], including over 15 studies of validated susceptibility loci with similar effect sizes; and we have previously replicated metabolomics findings between the two populations [25].Consequently despite the differences in study characteristics we consider GACRS a very strong replication population for CAMP.

In CAMP, metabolomic profiling was performed on serum, while in Costa Rica plasma was used. Nevertheless, metabolomics studies comparing results from plasma and serum show that although the specific metabolites may differ, the overall biological conclusions are likely to be the same [26]. It is therefore notable that we saw multiple cholesterol esters among the significantly interacting metabolites in both populations, although the actual metabolites were not always identical. It should also be noted that our conceptual model assumes circulating blood is a representative tissue for lung function. While other tissues such as airways brushing and bronchoalveolar lavage fluid, may be closer to the lung, metabolomics profiling of such biospecimens has been shown to be limited by issues such as contamination, dilution, lack of standardization and their invasive nature [27,28]. Mounting research demonstrates the suitability and success of blood omic-based lung disease studies [18,25,29,30,31,32]. Furthermore, blood is clinically relevant and readily accessible; vital for clinical translation which is the ultimate aim of many metabolomic studies of complex diseases.

Finally, Most of the metabolites reported as significant were not robust to correction for multiple testing according the Benjamini-Hochberg procedure [33]. However, there are currently no consensus standards for multiple testing correction in metabolomics; most commonly applied correction procedures are considered too stringent. This is due to the existence of metabolites within regulated connected biological pathways; the metabolites comprising these pathways, particularly those involved in the same biochemical reactions, are highly correlated and therefore cannot be considered truly independent. Thus, we considered a liberal *p*-value threshold whilst also reporting the FDR-corrected results. Finally, the beta coefficients for the change in BDR were somewhat modest, and further work is needed to consider potential clinical utility.

## 4. Materials and Methods

### 4.1. Study Population

Statistical analyses were first conducted in CAMP [34], then replication performed in GACRS [35]. Both study populations have previously been described.

#### 4.1.1. Discovery Population

CAMP [34] is a multi-center, randomized, double-masked, clinical trial designed to determine the long-term effects of different treatment regimens for mild to moderate asthma in children (ClinicalTrials.gov Identifier: NCT00000575). CAMP enrolled 1041 children aged 5 to 13 years at baseline between December 1993 and September 1995. All children completed a protocol including questionnaires, spirometry, and collection of blood, and were followed up for an average of 16 years. CAMP was approved by the institutional review board of Partners Healthcare (Protocol#: 1999-P-001549/29), by the CAMP clinical and Data Coordinating centers. Each child provided assent and their parent/guardian signed a consent statement.

#### 4.1.2. Replication Population

The GACRS [25] recruited a total of 1165 children aged 6–4 years with asthma from the Central Valley of Costa Rica between February 2001 and August 2008. At enrollment, all children completed a similar protocol to that in CAMP, including spirometry and blood collection. Written parental and child consent was obtained. The study was approved by the Partners Human Research Committee at Brigham and Women’s Hospital (USA); (Protocol#: 2000-P-001130/55), and the Ethics Committee of the Hospital Nacional de Niños (Costa Rica).

### 4.2. Spirometry

Lung function was measured at time-points concurrent to (i) study baseline, (ii) end-point (~four years post-baseline) and (iii) follow-up (~ten years post-baseline) blood draws in CAMP, and at the recruitment blood draw in GACRS. Lung function was measured by spirometry using a Survey Tach Spirometer (Warren E. Collins; Braintree, MA) in accordance with American Thoracic Society recommendations (eMethods). BDR was defined as the best forced expiratory volume in the 1st second (FEV_1_) post-bronchodilator minus FEV_1_ pre-bronchodilator, divided by FEV_1_ pre-bronchodilator (expressed as percentage).

### 4.3. Metabolomic Profiling

Metabolomic profiling was performed on serum samples in CAMP (500 µL) and on plasma samples in the GACRS (30 µL). In both cases, blood was shipped to the sample repository to the Broad Institute (Cambridge, MA, USA) on dry ice for metabolomic profiling. Samples were thawed on ice for sub-aliquoting for each of the metabolomic methods, and then re-frozen on dry ice and stored at −80 °C until analysis.

Methods have been described previously (full details are provided in eMethods). In brief, in CAMP four complimentary liquid chromatography tandem mass spectrometry (LC-MS) methods were used. (i) Hydrophilic interaction liquid chromatography (HILIC) analyses of water soluble metabolites in the negative ionization mode (HILIC-neg) [36]; MS analyses were carried out using electrospray ionization and selective multiple reaction monitoring scans in the negative ion mode. To create the method, de-clustering potentials and collision energies were optimized for each metabolite by infusion of reference standards (ii) HILIC analyses of water soluble metabolites in the positive ionization mode (HILIC-pos) [37,38,39,40]; MS analyses were carried out using electrospray ionization in the positive ion mode using full scan analysis over 70–800 m/z at 70,000 resolution and 3 Hz data acquisition rate (iii) Positive ion mode analyses of polar and non-polar plasma lipids (C8-pos) [37,39,40]; MS analyses were carried out using electrospray ionization in the positive ion mode using full scan analysis over 200–1000 m/z at 70,000 resolution and 3 Hz data acquisition rate. Lipid identities were determined based on comparison to reference plasma extracts and were denoted by total number of carbons in the lipid acyl chain(s) and total number of double bonds in the lipid acyl chain(s). (iv) Negative ion mode analyses of free fatty acids and bile acids (C18-neg) were conducted using an LC-MS system with samples prepared using solid phase extraction. MS analyses were carried out in the negative ion mode using electrospray ionization, full scan MS acquisition over 70–850 m/z, and a resolution setting of 70,000. Metabolite identities were confirmed using authentic reference standards.

In GACRS, the same four methods were used to profile the plasma samples, however solid phase extraction was not performed for the C18-neg platform.

To evaluate data quality and enable standardization of data across the analytical queue and among batches, pooled serum reference samples were analyzed after intervals of 20 study samples. Results for each metabolite were standardized using the ratio of the value of the sample to the value of the nearest pooled reference multiplied by the median of all reference values for the metabolite. Raw data from Q Exactive/Exactive Plus instruments were processed using TraceFinder 3.3 software (Thermo Fisher Scientific; Waltham, MA) and Progenesis QI (Nonlinear Dynamics; Newcastle upon Tyne, UK) while MultiQuant 2.1 (SCIEX; Framingham, MA) was used to process 5500 QTRAP data. Compounds were identified by their exact mass and by matching their retention times to authentic reference standards/reference. In many cases, isomeric compounds were analyzed and in cases where the compound could not be resolved by chromatography, a general name for the compound is reported (e.g., pentose phosphate for ribulose 5-phosphate/ribose 5-phosphate). Only identified metabolites are included in these analyses.

Quality control (QC) was performed using previously described methods [25]: Metabolite features with a signal-to-noise ratio <10 and/or features with undetectable/missing levels for >10% of samples were excluded. Remaining missing values were imputed with the median peak intensity for that feature across the whole population. Features with a coefficient of variance in the QC samples > 25% were excluded to ensure technical reproducibility.

Metabolites were analyzed as measured LC-MS peak areas, and log10-transformed and *pareto* scaled prior to analysis. After QC and data processing a total of 501 named metabolites were identified in CAMP and 615 were identified in GACRS; 430 were common to the two populations.

### 4.4. Statistical Analysis

In CAMP, participants had BDR and metabolite measurements at one to three time points: (i) study baseline, (ii) end-point and/or (iii) follow-up visits. Using linear mixed models to account for multiple measures per subject, we fit 501 random intercept models to test for an age by metabolite interaction on BDR for each metabolite. We included race, sex, clinic, treatment group, age, metabolite, and the interaction term age*metabolite for the fixed effects. For replication in GACRS samples, only a single time-point was available. We considered a multivariable linear regression for BDR as a function of sex, race, age at sample, metabolite, and the interaction age*metabolite. We performed sensitivity analyses for those significant metabolites in CAMP that replicated in the GACRS, to explore the role of race and gender in the results. The Benjamini-Hochberg procedure [33] was utilized to generate False Discovery Rate (FDR) corrected *p*-values, using the function ‘p.adjust’ from the R package ‘Stats’.

With a focus on those metabolites that were nominally significant (*p* < 0.05) in both populations, we visualized the age by metabolite interactions by plotting the predicted relationship between BDR and age at the 25th, 50th and 75th percentile level of the relevant metabolite. Plots of the raw data for the relationship between BDR and age are given in the Supplementary Figures S7–S13.

All models were run in R (v3.4.0) using the packages ‘effects’ and the ‘lme’ function in the ‘nlme’ package. We also conducted power analyses for the main results presented here in the Supplementary Material. The R code used to conduct these analyses is publicly available on GitHub (https://github.com/SharonLutz/ePowerLI). A QQ plot for the age by metabolite interaction on BDR in the CAMP study is given in the Supplementary Figure S16.

## 5. Conclusions

In this study, we identified seven metabolites that demonstrated interactions with age on BDR, and which may represent targets for therapeutic or preventative interventions. This represents the first study to consider the role of age*metabolite interactions in lung function and respiratory health, providing new insights into the underlying biology of age-specific responses to therapeutics in asthma.

## Figures and Tables

**Table 1:** Characteristics of the CAMP participants across three time-points with serum metabolomic. profiling.

		Baseline (n = 560)		Study End (n = 563)		Follow-up (n = 295)	
Characteristic		n	%	n	%	n	%
**Sex**	**Male**	359	64.1%	357	63.4%	189	64.1%
	**Female**	201	35.9%	206	36.6%	106	35.9%
**Race**	**White**	395	70.5%	399	70.9%	213	72.2%
	**Black**	82	14.6%	81	14.4%	35	11.9%
	**Hispanic**	56	10.0%	56	9.9%	21	7.1%
	**Other**	27	4.8%	27	4.8%	26	8.8%
**Treatment Group**	**Budesonide**	151	27.0%	156	27.7%	78	26.4%
	**Nedocromil**	171	30.5%	169	30.0%	83	28.1%
	**Placebo**	238	42.5%	238	42.3%	134	45.4%
**Age at blood sample**	**mean (SD) [range]**	8.8 (2.1)	[5.1, 13.2]	12.8 [2.2]	[9.1, 17.2]	16.8 (2.9)	[12.2, 25.9]
**BDR at blood sample**	**mean (SD) [range]**	0.11 (0.10)	[−0.17,0.82]	0.09 [0.08]	[−0.08, 0.59]	0.08 (0.07)	[−0.14,0.49]
**Other available time-points**	**Baseline**	-	-	558	99.1%	294	99.7%
	**End**	558	99.6%	-	-	295	100.0%
	**Follow-up**	294	52.5%	295	52.4%	-	-

SD—Standard deviation; BDR—Bronchodilator Response.

**Table 2:** Metabolites significantly interacting with age in BDR in CAMP.

Metabolite	Beta	Interaction *p*-Value	Interaction *q*-Value ^a^
2-hydroxyglutarate	−0.004	1.77 × 10^−4^	0.089
adipate	−0.004	0.001	0.136
GABA	0.004	0.004	0.468
2-O-methyladenosine	0.002	0.005	0.468
3-methyladipate/pimelate	−0.002	0.005	0.468
C18:1 CE	0.005	0.006	0.468
ectoine	−0.002	0.007	0.468
saccharin	0.001	0.008	0.468
C18:3 CE	0.004	0.010	0.468
sebacate	−0.002	0.011	0.468
suberate	−0.002	0.011	0.468
C36:1 DAG	−0.002	0.011	0.468
linoleoyl ethanolamide	0.002	0.012	0.477
C18:0 CE	0.004	0.014	0.489
C22:5 CE	0.003	0.015	0.492
C16:0 CE	0.005	0.021	0.576
cortisone	0.002	0.022	0.576
C54:1 TAG	−0.002	0.022	0.576
C10:2 carnitine	−0.001	0.024	0.576
arginine	0.004	0.024	0.576
C6 carnitine	0.002	0.025	0.576
taurodeoxycholate/taurochenodeoxycholate	−0.002	0.026	0.576
C56:2 TAG	−0.003	0.027	0.576
C36:0 DAG	−0.004	0.028	0.576
C30:0 DAG	−0.001	0.029	0.589
C36:2 DAG or TAG fragment	−0.002	0.032	0.614
C58:10 TAG	0.001	0.038	0.622
sphingosine	0.002	0.039	0.622
C36:2 DAG	−0.002	0.041	0.622
C20:3 CE	0.003	0.042	0.622
phenyllactate	−0.003	0.042	0.622
C20:4 CE	0.003	0.043	0.622
C32:1 DAG	−0.002	0.043	0.622
C5 carnitine	0.002	0.043	0.622
C54:2 TAG	−0.002	0.044	0.622
C16:1 CE	0.003	0.045	0.622
ribothymidine	0.002	0.046	0.622
taurocholate	−0.002	0.047	0.622
C3 carnitine	0.002	0.050	0.641

*GABA—Gamma-Aminobutyric acid;CE—Cholesterol Ester;DAG—Diacylglycerol;TAG-Triacylglycerol.***^a^** Computed according to the Benjamini Hochberg Procedure.

**Table 3:** Metabolites that had a significant interaction with age in the determination of BDR in CAMP and their interaction effect in the replication cohort, GACRS. *Only the 29 metabolites (of 38 significant) that were measured in GACRS are shown*.

Metabolite	CAMP			GACRS		
	Beta	Interaction *p*-Value	Interaction *q*-Value ^a^	Beta	Interaction *p*-Value	Interaction *q*-Value ^a^
2-hydroxyglutarate *	−0.004	1.80 × 10^−4^	0.089	−0.015	0.018	0.997
GABA**^.^**	0.004	0.004	0.468	0.01	0.085	0.997
3-methyladipate/pimelate	−0.002	0.005	0.468	−0.01	0.133	0.997
C18:1 CE *	0.005	0.006	0.468	0.023	0.041	0.997
C18:3 CE	0.004	0.01	0.468	0.009	0.203	0.997
C36:1 DAG	−0.002	0.011	0.468	0.001	0.823	0.997
linoleoyl ethanolamide	0.002	0.012	0.477	0.001	0.807	0.997
C18:0 CE**^.^**	0.004	0.014	0.489	0.012	0.101	0.997
C22:5 CE	0.003	0.015	0.492	0.011	0.125	0.997
C16:0 CE**^.^**	0.005	0.021	0.576	0.023	0.056	0.997
Cortisone	0.002	0.022	0.576	0.001	0.812	0.997
C54:1 TAG	−0.002	0.022	0.576	−0.001	0.760	0.997
C10:2 carnitine	−0.001	0.024	0.576	−0.002	0.683	0.997
Arginine	0.004	0.024	0.576	−0.002	0.747	0.997
C6 carnitine	0.002	0.025	0.576	−0.001	0.841	0.997
taurodeoxycholate/taurochenodeoxycholate	−0.002	0.026	0.576	−0.004	0.321	0.997
C56:2 TAG	−0.003	0.027	0.576	−0.003	0.572	0.997
C30:0 DAG	−0.001	0.029	0.589	0.001	0.799	0.997
C58:10 TAG	0.001	0.038	0.622	−0.003	0.572	0.997
C36:2 DAG	−0.002	0.04	0.622	0.001	0.949	0.997
C20:3 CE	0.003	0.042	0.622	0.01	0.189	0.997
C20:4 CE**^.^**	0.003	0.043	0.622	0.017	0.076	0.997
C32:1 DAG	−0.002	0.043	0.622	0.001	0.832	0.997
C5 carnitine	0.002	0.043	0.622	−0.001	0.861	0.997
C54:2 TAG	−0.002	0.044	0.622	−0.002	0.760	0.997
C16:1 CE	0.003	0.045	0.622	0.01	0.195	0.997
Ribothymidine**^.^**	0.002	0.046	0.622	0.01	0.088	0.997
Taurocholate	−0.002	0.047	0.622	−0.005	0.166	0.997
C3 carnitine	0.002	0.05	0.641	0.003	0.629	0.997

GABA—*Gamma-Aminobutyric acid; CE—Cholesterol Ester; DAG—Diacylglycerol; TAG-Triacylglycerol.* * Significant (*p* < 0.05) replication between CAMP and GACRS. Nominally significant (*p* < 0.1) replication between CAMP and GACRS. **^a^** *Computed according to the Benjamini Hochberg Procedure*.

**Table 4:** Relationship between metabolite levels and age at sample collection in CAMP and GACRS.

Metabolite	CAMP												Costa Rica			
	Baseline				Study End				Follow-up							
	Beta	95% CI	*p-Value*	*q-Value ^a^*	Beta	95% CI	*p-Value*	*q-Value ^a^*	Beta	95% CI	*p-Value*	*q-Value ^a^*	Beta	95% CI	*p-Value*	*q-Value ^a^*
**2-hydroxy** **glutarate**	−0.015	(−0.034, 0.004)	*0.114*	*0.314*	−0.013	(−0.029, 0.002)	*0.082*	*0.082*	0.032	(0.014, 0.05)	0.001 *	*0.001*	−0.008	(−0.038, 0.021)	*0.581*	*0.931*
**C18:1 CE**	−0.008	(−0.02, 0.003)	*0.159*	*0.314*	−0.017	(−0.028, −0.007)	0.001 *	*0.009*	−0.02	(−0.032, −0.008)	0.002 *	*0.002*	−0.005	(−0.022, 0.011)	*0.52*	*0.931*
**C16:0 CE**	9.5 × 10^−5^	(−0.01, 0.011)	*0.986*	*0.986*	−0.012	(−0.022, −0.003)	0.014 *	*0.016*	−0.009	(−0.02, 0.002)	0.108 *	*0.126*	−0.008	(−0.024, 0.008)	*0.317*	*0.921*
**GABA**	−0.012	(−0.028, 0.004)	*0.132*	*0.314*	−0.019	(−0.034, −0.004)	0.012 *	*0.016*	−0.029	(−0.046, −0.013)	0.001 *	*0.001*	−0.008	(−0.038, 0.021)	*0.581*	*0.931*
**C18:0 CE**	3.7 × 10^−4^	(−0.014, 0.014)	*0.959*	*0.986*	−0.018	(−0.031, −0.005)	0.005 *	*0.012*	−0.036	(−0.051, −0.02)	*7.7* × *10*^−6^ *	*2.7* × *10*^−5^	0.006	(−0.017, 0.029)	*0.625*	*0.931*
**C20:4 CE**	−0.009	(−0.021, 0.004)	*0.18*	*0.314*	−0.018	(−0.031, −0.006)	0.005 *	*0.012*	−0.006	(−0.021, 0.008)	*0.404*	*0.404*	−0.012	(−0.032, 0.009)	*0.265*	*0.921*
**Ribo** **thymidine**	−0.009	(−0.025, 0.006)	*0.236*	*0.330*	−0.018	(−0.031, −0.005)	0.007 *	*0.012*	−0.062	(−0.077, −0.047)	*6.2 × 10*^−15^ *	*4.3* × *10*^−14^	4.7 × 10^−4^	(−0.031, 0.032)	*0.977*	*0.972*

GABA—Gamma-Aminobutyric acid; CE—Cholesterol Ester. * Significant at the95% confidence interval. *^a^* *Computed according to the Benjamini-Hochberg procedure.*

## Supplementary Materials

The following are available online at https://www.mdpi.com/2218-1989/9/9/179/s1, Supplementary Methods; Table S1: Characteristics of the GACRS participants with plasma metabolomic profiling; Table S2: Metabolites significantly interacting with age in BDR in GACRS; Table S3: Metabolites significantly interacting with age in BDR in GACRS with adjustment for ancestry score Table S4: Metabolites significantly interacting with age in BDR stratified by race in the CAMP population. Table S5: Sex Stratified Age*metabolite interactions for BDR outcome in the CAMP cohort; Table S6: Sex Stratified Age*metabolite interactions for BDR outcome in the GACRS population; Figure S1: Correlation between 39 metabolites that interact with Age in BDR measured at baseline in CAMP; Figure S2: Correlation between 39 metabolites that interact with Age in BDR measured at study end point in CAMP; Figure S3: Correlation between 39 metabolites that interact with Age in BDR measured at study follow-up in CAMP Figure S4: Relationship between Age and BDR stratified by plasma levels of 2-hydroxyglutarate in GACRS; Figure S5: Relationship between Age and BDR stratified by plasma levels of GABA; ribothymidine C18:1 CE; C16:0 CE; C18:0 CE; C20:4 CE in GACRS Figure S6: Relationship between Age and BDR stratified by plasma levels of 2-hydroxyglutarate in males and in females from CAMP Figures S7–S13: Raw data for the relationship between BDR and age stratified by levels of the given metabolite. A description of the power analysis is given along with corresponding Figures S14 and S15. Figure S16: QQ plot for the age by metabolite interaction on BDR in the CAMP study.
