# Supplementary material for: Pharmacometabolomics of Bronchodilator Response in Asthma and the Role of Age-Metabolite Interactions
Source: Metabolites. 2019 Sep 7;9(9):179. doi: 10.3390/metabo9090179 (PMC6780678; doi:10.3390/metabo9090179)
Supplement: Supplementary file 1 [file metabolites-09-00179-s001.zip › metabolites-559853-supplementary.docx]

**SUPPLEMENTAL MATERIALS**

**Pharmacometabolomics of Bronchodilator Response in Asthma and the role of Age-metabolite interactions**

*Rachel S. Kelly, PhD^1*^, Joanne E. Sordillo ScD^2*^, Sharon M. Lutz PhD^2^, Lydiana Avila MD^3^, Manuel Soto-Quiros MD, PhD^3^, Juan C. Celedón MD ^4^, Michael J. McGeachie PhD, ^1^, Amber Dahlin PhD^1^, Kelan Tantisira MD^1^, Mengna Huang^1^, Clary Clish PhD^5^, Scott T Weiss MD^1^, Jessica Lasky-Su ScD^1^^, Ann Chen Wu MD^2^^*

*^1^ Channing Division of Network Medicine, Brigham and Women’s Hospital, Harvard Medical School, 181 Longwood Avenue, Boston MA 02115*

*^2^PRecisiOn Medicine Translational Research (PROMoTeR) Center, Department of Population Medicine, Harvard Medical School and Harvard Pilgrim Health Care, Boston, MA*

*^3^Department of Pediatrics, Hospital Nacional de Niños, San José, Costa Rica*

*^4^Division of Pediatric Pulmonary Medicine, UPMC Children’s Hospital of Pittsburgh, University of Pittsburgh*

*5 The Broad Institute, Cambridge, MA*

**Supplementary Methods**

**Spirometry**

Lung function was measured by spirometry using a Survey Tach Spirometer (Warren E. Collins; Braintree, MA) in accordance with the American Thoracic Society recommendations. Prior to assessment, the children were told to withhold short-acting bronchodilators for at least 4 hours. Spirometric maneuvers were conducted with the children seated and wearing a nose clip. The measurements were calibrated for sex, age and height according to reference values. After completing baseline spirometry, the children were given 200 μg (2 puffs) of an albuterol pressurized metered-dose inhaler (pMDI) using a spacer device. Spirometry was repeated after 15 min.

**Metabolomic Profiling in CAMP**

Serum metabolites were profiled using a combination of using four complimentary liquid chromatography tandem mass spectrometry (LC-MS) methods. Hydrophilic interaction liquid chromatography (HILIC) analyses of water soluble metabolites in the negative ionization mode (HILIC-neg) were conducted using an LC-MS system comprised of an AQUITY UPLC system (Waters; Milford, MA and a 5500 QTRAP mass spectrometer (SCIEX; Framingham, MA) as described previously^1^. Briefly, serum samples (30 µL) were prepared via protein precipitation with the addition of four volumes of 80% methanol containing inosine-15N4, thymine-d4 and glycocholate-d4 internal standards (Cambridge Isotope Laboratories; Andover, MA). The samples were centrifuged (10 min, 9,000 x g, 4°C), and the supernatants were injected directly onto a 150 x 2.0 mm Luna NH2 column (Phenomenex; Torrance, CA). The column was eluted at a flow rate of 400 µL/min with initial conditions of 10% mobile phase A (20 mM ammonium acetate and 20 mM ammonium hydroxide in water) and 90% mobile phase B (10 mM ammonium hydroxide in 75:25 v/v acetonitrile/methanol) followed by a 10 min linear gradient to 100% mobile phase A. MS analyses were carried out using electrospray ionization and selective multiple reaction monitoring scans in the negative ion mode. To create the method, declustering potentials and collision energies were optimized for each metabolite by infusion of reference standards. The ion spray voltage was -4.5 kV and the source temperature was 500°C.

HILIC analyses of water soluble metabolites in the positive ionization mode (HILIC-pos) were conducted using an LC-MS system comprised of a Shimadzu Nexera X2 U-HPLC (Shimadzu Corp.; Marlborough, MA) coupled to a Q Exactive hybrid quadrupole orbitrap mass spectrometer (Thermo Fisher Scientific; Waltham, MA) as described previously ^2-5^. Briefly, serum samples (10 µL) were prepared via protein precipitation with the addition of nine volumes of 74.9:24.9:0.2 v/v/v acetonitrile/methanol/formic acid containing stable isotope-labeled internal standards (valine-d8, Sigma-Aldrich; St. Louis, MO; and phenylalanine-d8, Cambridge Isotope Laboratories; Andover, MA). The samples were centrifuged (10 min, 9,000 x g, 4°C), and the supernatants were injected directly onto a 150 x 2 mm, 3 µm Atlantis HILIC column (Waters; Milford, MA). The column was eluted isocratically at a flow rate of 250 µL/min with 5% mobile phase A (10 mM ammonium formate and 0.1% formic acid in water) for 0.5 minute followed by a linear gradient to 40% mobile phase B (acetonitrile with 0.1% formic acid) over 10 minutes. MS analyses were carried out using electrospray ionization in the positive ion mode using full scan analysis over 70-800 m/z at 70,000 resolution and 3 Hz data acquisition rate. Other MS settings were: sheath gas 40, sweep gas 2, spray voltage 3.5 kV, capillary temperature 350°C, S-lens RF 40, heater temperature 300°C, microscans 1, automatic gain control target 1e6, and maximum ion time 250 ms.

Positive ion mode analyses of polar and non-polar plasma lipids (C8-pos) were conducted using an LC-MS system comprised of a Shimadzu Nexera X2 U-HPLC (Shimadzu Corp.; Marlborough, MA) coupled to a Exactive Plus orbitrap mass spectrometer (Thermo Fisher Scientific; Waltham, MA) as described previously ^2,4,5^. Serum samples (10 µL) were extracted for lipid analyses using 190 µL of isopropanol containing 1,2-didodecanoyl-sn-glycero-3-phosphocholine (Avanti Polar Lipids; Alabaster, AL). After centrifugation, supernatants were injected directly onto a 100 x 2.1 mm, 1.7 µm ACQUITY BEH C8 column (Waters; Milford, MA). The column was eluted isocratically with 80% mobile phase A (95:5:0.1 vol/vol/vol 10mM ammonium acetate/methanol/formic acid) for 1 minute followed by a linear gradient to 80% mobile-phase B (99.9:0.1 vol/vol methanol/formic acid) over 2 minutes, a linear gradient to 100% mobile phase B over 7 minutes, then 3 minutes at 100% mobile-phase B. MS analyses were carried out using electrospray ionization in the positive ion mode using full scan analysis over 200–1000 m/z at 70,000 resolution and 3 Hz data acquisition rate. Other MS settings were: sheath gas 50, in source CID 5 eV, sweep gas 5, spray voltage 3 kV, capillary temperature 300°C, S-lens RF 60, heater temperature 300°C, microscans 1, automatic gain control target 1e6, and maximum ion time 100 ms. Lipid identities were determined based on comparison to reference plasma extracts and were denoted by total number of carbons in the lipid acyl chain(s) and total number of double bonds in the lipid acyl chain(s).

Negative ion mode analyses of free fatty acids and bile acids (C18-neg) were conducted using an LC-MS system comprised of a Shimadzu Nexera X2 U-HPLC (Shimadzu Corp.; Marlborough, MA) coupled to a Q Exactive hybrid quadrupole orbitrap mass spectrometer (Thermo Fisher Scientific; Waltham, MA). Samples were prepared using solid phase extraction. Briefly, 1.5 mL of methanol containing 1 ng/mL 15R-15-methyl PGA_2_, 15S-15-methyl PGD_2_, and 15S-15-methyl PGE_2_ internal standards was added to 500 µL serum. The samples were centrifuged (9000 x g, 4°C, 10 minutes) and the supernatants were collected and diluted to 10 mL using water. Samples were acidified to pH 3 using 1 M hydrochloric acid and loaded onto pre-conditioned Oasis HLB solid phase extraction cartridges (12cc, 500 mg; Waters; Milford MA). The cartridges were washed with 8 mL of water and metabolites were eluted using 8 mL of methanol. Samples were dried using a TurboVap LV (Biotage) and re-suspended in 100 µL of 80% methanol containing 15R-15-methyl-PGF_2alpha_ internal standard. Extracts (10 µL) were injected onto a 150 x 2.1 mm ACQUITY BEH C18 column (Waters; Milford, MA). The column was eluted isocratically at a flow rate of 450 µL/min with 80% mobile phase A (0.01% formic acid in water) for 3 minutes followed by a linear gradient to 100% mobile phase B (0.01% acetic acid in acetonitrile) over 12 minutes. MS analyses were carried out in the negative ion mode using electrospray ionization, full scan MS acquisition over 70-850 m/z, and a resolution setting of 70,000. Metabolite identities were confirmed using authentic reference standards. Other MS settings were: sheath gas 45, sweep gas 5, spray voltage -3.5 kV, capillary temperature 320°C, S-lens RF 60, heater temperature 300°C, microscans 1, automatic gain control target 1e6, and maximum ion time 250 ms.

**Metabolomic Profiling in GACRS**

Plasma metabolites were measured using four distinct liquid chromatography tandem mass spectrometry (LC-MS) methods designed to measure complementary sets of metabolites: polar metabolites measured in the positive ion mode (HILIC-positive), polar metabolites measured in the negative ion mode (HILIC-negative), metabolites of intermediate polarity (e.g. free fatty acids and bile acids; C18-negative), and lipids (C8-positive). Briefly, hydrophilic interaction liquid chromatography (HILIC) analyses of water soluble metabolites in the positive ionization mode were conducted using an LC-MS system comprised of a Shimadzu Nexera X2 U-HPLC (Shimadzu Corp.; Marlborough, MA) coupled to a Q Exactive hybrid quadrupole orbitrap mass spectrometer (Thermo Fisher Scientific; Waltham, MA). Plasma samples (10 µL) were extracted using 90 µL of 74.9:24.9:0.2 v/v/v acetonitrile/methanol/formic acid containing stable isotope-labeled internal standards (valine-d8, Sigma-Aldrich; St. Louis, MO; and phenylalanine-d8, Cambridge Isotope Laboratories; Andover, MA). The samples were centrifuged (10 min, 9,000 x g, 4°C), and the supernatants were injected directly onto a 150 x 2 mm, 3 µm Atlantis HILIC column (Waters; Milford, MA). The column was eluted isocratically at a flow rate of 250 µL/min with 5% mobile phase A (10 mM ammonium formate and 0.1% formic acid in water) for 0.5 minute followed by a linear gradient to 40% mobile phase B (acetonitrile with 0.1% formic acid) over 10 minutes. MS analyses were carried out using electrospray ionization in the positive ion mode using full scan analysis over 70-800 m/z at 70,000 resolution and 3 Hz data acquisition rate. Other MS settings were: spray voltage 3.5 kV, capillary temperature 350°C, and heater temperature 300°C. HILIC analyses of water soluble metabolites in the negative ionization mode were conducted using an LC-MS system comprised of an AQUITY UPLC system (Waters; Milford, MA and a 5500 QTRAP mass spectrometer (SCIEX; Framingham, MA). Plasma samples (30 µL) were extracted using 120 µL of 80% methanol containing inosine-15N4, thymine-d4 and glycocholate-d4 internal standards (Cambridge Isotope Laboratories; Andover, MA). The samples were centrifuged (10 min, 9,000 x g, 4°C), and the supernatants were injected directly onto a 150 x 2.0 mm Luna NH2 column (Phenomenex; Torrance, CA). The column was eluted at a flow rate of 400 µL/min with initial conditions of 10% mobile phase A (20 mM ammonium acetate and 20 mM ammonium hydroxide in water) and 90% mobile phase B (10 mM ammonium hydroxide in 75:25 v/v acetonitrile/methanol) followed by a 10 min linear gradient to 100% mobile phase A. MS analyses were carried out using electrospray ionization and selective multiple reaction monitoring scans in the negative ion mode. To create the method, declustering potentials and collision energies were optimized for each metabolite by infusion of reference standards. The ion spray voltage was -4.5 kV and the source temperature was 500°C. Reversed-phase C18 chromatography/negative ion mode MS analyses of free fatty acids and bile acids were conducted using an LC-MS system comprised of a Shimadzu Nexera X2 U-HPLC (Shimadzu Corp.; Marlborough, MA) coupled to a Q Exactive hybrid quadrupole orbitrap mass spectrometer (Thermo Fisher Scientific; Waltham, MA). Plasma samples (30 µL) were extracted using 90 uL of methanol containing PGE_2_-d4 (Cayman Chemical Co.; Ann Arbor, MI) and centrifuged (10 min, 9,000 x g, 4°C). The samples were injected onto a 150 x 2 mm ACQUITY T3 column (Waters; Milford, MA). The column was eluted isocratically at a flow rate of 400 µL/min with 60% mobile phase A (0.1% formic acid in water) for 4 minutes followed by a linear gradient to 100% mobile phase B (acetonitrile with 0.1% formic acid) over 8 minutes. MS analyses were carried out in the negative ion mode using electrospray ionization, full scan MS acquisition over 200-550 m/z, and a resolution setting of 70,000. Metabolite identities were confirmed using authentic reference standards. Other MS settings were: spray voltage -3.5 kV, capillary temperature 320°C, and heater temperature 300°C. C8 chromatography analyses of polar and non-polar plasma lipids were conducted using an LC-MS system conducted using an LC-MS system comprised of a Shimadzu Nexera X2 U-HPLC (Shimadzu Corp.; Marlborough, MA) coupled to a Exactive Plus orbitrap mass spectrometer (Thermo Fisher Scientific; Waltham, MA). Plasma samples (10 µL) were extracted using 190 µL of isopropanol containing 1,2-didodecanoyl-sn-glycero-3-phosphocholine (Avanti Polar Lipids; Alabaster, AL). After centrifugation, supernatants were injected directly onto a 100 x 2.1 mm, 1.7 µm ACQUITY BEH C8 column (Waters; Milford, MA). The column was eluted isocratically with 80% mobile phase A (95:5:0.1 vol/vol/vol 10mM ammonium acetate/methanol/formic acid) for 1 minute followed by a linear gradient to 80% mobile-phase B (99.9:0.1 vol/vol methanol/formic acid) over 2 minutes, a linear gradient to 100% mobile phase B over 7 minutes, then 3 minutes at 100% mobile-phase B. MS analyses were carried out using electrospray ionization in the positive ion mode using full scan analysis over 200–1000 m/z at 70,000 resolution and 3 Hz data acquisition rate. Other MS settings were: spray voltage 3 kV, capillary temperature 300°C, and heater temperature 300°C. Lipid identities were determined based on comparison to reference plasma extracts and were denoted by total number of carbons in the lipid acyl chain(s) and total number of double bonds in the lipid acyl chain(s).

To account for potential batch effect a cassette of two pooled plasma samples was run at intervals of 20 study samples. Each pooled plasma sample cassette included a pooled sample prepared from the study samples for data standardization using a "nearest neighbor" approach as well as a pooled plasma sample for determination of data quality and precision both before and after data standardization.

Raw data from Q Exactive/Exactive Plus MS systems were processed using TraceFinder software (Thermo Fisher Scientific; Waltham, MA) and Progenesis QI (Nonlinear Dynamics; Newcastle upon Tyne, UK). Raw data collected using the HILIC-negative method were processed using MultiQuant 2.1 software (SCIEX; Framingham, MA).

**Genotyping and Genotype Principal Components**

In the CAMP study participants were genotyped using the HumanHap550 Genotyping BeadChip or Infinium HD Human610-Quad BeadChip by Illumina, Inc (San Diego, CA). In GACRS genotyping was conducted using the Illumina HumanOmniExpress-12v1 platform by Illumina Inc. (San Diego CA)

Principal Components were calculated using [TRACE](http://www.google.com/url?q=http%3A%2F%2Fcsg.sph.umich.edu%2Fchaolong%2FLASER%2F&sa=D&sntz=1&usg=AFQjCNGMLmQzKwAXtmChg_liICS-xZuYBQ) (fasT and Robust Ancestry Coordinate Estimation, Version 1.01)  TRACE was run with DIM=20, and default values for all other parameters. The input genotype dataset for CAMP had 518111 markers from various chip arrays. Hapmap3_r2_b36 was used as a reference with 1440616 markers. Input data were prepared for TRACE as follows: 1) Markers in long-range LD were removed (28 chromosomal ranges, 11067 markers total) 2) Markers not shared with the Hapmap3 reference were removed 3) Markers with a minor allele frequency <= 0.05 were removed 4) PLINK ([https://www.cog-genomics.org/plink2](https://www.google.com/url?q=https%3A%2F%2Fwww.cog-genomics.org%2Fplink2&sa=D&sntz=1&usg=AFQjCNH4URWyqPGFn7eRUi1fhzVIh4KDug), v1.90b3.29) was run in --indep-pairwise mode with window-size=1500, step-size=50, and r^2 threshold=0.1, and markers in the *[prune.in](http://www.google.com/url?q=http%3A%2F%2Fprune.in%2F&sa=D&sntz=1&usg=AFQjCNEItsA9RNHl6bmKNPyT9-xtavKlTQ) file were kept.

**Supplementary Tables**

**Table S1:** Characteristics of the GACRS participants with plasma metabolomic profiling

|  | | **Total Population** | | **Males** | | **Females** | |
| --- | --- | --- | --- | --- | --- | --- | --- |
|  |  | **n** | **%** | **n** | **%** | **n** | **%** |
| **Gender** | **Male** | 185 | 57.80% | 185 | 100% | - | - |
|  | **Female** | 135 | 42.20% | - | - | 135 | 100% |
| **Age at recruitment** | **mean (SD) [range]** | 9.1 (1.7) | [6.0, 13.3] | 9.2 (1.8) | [6.0, 13.3] | 9.0 (1.7) | [6.1, 13.0] |
| **BDR at recruitment** | **mean (SD) [range]** | 0.05 (0.09) | [-0.16,0.47] | 0.05 (0.09) | [-0.16,0.47] | 0.05 (0.08) | [-0.08,0.37] |

*SD- Standard deviation*

*BDR – Bronchodilator Response*

**Table S2:** Metabolites significantly interacting with age in BDR in GACRS (p<0.05)

| **Metabolite** | **Beta** | **Interaction p-value** | **Interaction q-value** |
| --- | --- | --- | --- |
| C34:5 PC plasmalogen | 0.013 | 0.006 | 0.997 |
| methylimidazoleacetic acid | 0.014 | 0.007 | 0.997 |
| tyramine | -0.030 | 0.008 | 0.997 |
| C20:1 CE | 0.016 | 0.011 | 0.997 |
| C22:4 CE | 0.012 | 0.014 | 0.997 |
| PE-plasmalogen C37:4 | 0.017 | 0.017 | 0.997 |
| C19:1 CE | 0.015 | 0.017 | 0.997 |
| biliverdin | 0.011 | 0.018 | 0.997 |
| 2-hydroxyglutarate | -0.015 | 0.018 | 0.997 |
| 1-methylnicotinamide | 0.014 | 0.023 | 0.997 |
| C18:1 CE | 0.023 | 0.041 | 0.997 |
| C17:1 CE | 0.014 | 0.047 | 0.997 |

*CE – Cholesterol Ester*

**Table S3**. Metabolites significantly interacting with age in BDR with adjustment for ancestry score in the CAMP population

| **Metabolite** | **Adjusting For Race**^a^ | | | **Adjusting for Ancestry Score**^b^ | | |
| --- | --- | --- | --- | --- | --- | --- |
|  | **Beta** | **Interaction p-value** | **Interaction q-value^d^** | **Beta** | **Interaction p-value** | **Interaction q-value^d^** |
| 2-hydroxyglutarate | -0.004 | 1.77x10^-4^ | 0.089 | -0.004 | 0.001 | 0.092 |
| adipate | -0.004 | 0.001 | 0.136 | -0.004 | 4.22x10^-04^ | 0.092 |
| GABA | 0.004 | 0.004 | 0.468 | 0.004 | 0.005 | 0.167 |
| 2-O-methyladenosine | 0.002 | 0.005 | 0.468 | 0.001 | 0.035 | 0.290 |
| 3-methyladipate/pimelate | -0.002 | 0.005 | 0.468 | -0.002 | 0.016 | 0.216 |
| C18:1 CE | 0.005 | 0.006 | 0.468 | 0.006 | 0.001 | 0.092 |
| ectoine | -0.002 | 0.007 | 0.468 | -0.002 | 0.023 | 0.219 |
| saccharin | 0.001 | 0.008 | 0.468 | 0.001 | 0.018 | 0.216 |
| C18:3 CE | 0.004 | 0.01 | 0.468 | 0.005 | 0.001 | 0.105 |
| sebacate | -0.002 | 0.011 | 0.468 | -0.002 | 0.039 | 0.298 |
| suberate | -0.002 | 0.011 | 0.468 | -0.002 | 0.021 | 0.217 |
| C36:1 DAG | -0.002 | 0.011 | 0.468 | -0.003 | 0.001 | 0.105 |
| linoleoyl ethanolamide | 0.002 | 0.012 | 0.477 | 0.002 | 0.007 | 0.182 |
| C18:0 CE | 0.004 | 0.014 | 0.489 | 0.004 | 0.013 | 0.216 |
| C22:5 CE | 0.003 | 0.015 | 0.492 | 0.004 | 0.002 | 0.112 |
| C16:0 CE | 0.005 | 0.021 | 0.576 | 0.006 | 0.002 | 0.112 |
| cortisone | 0.002 | 0.022 | 0.576 | 0.002 | 0.017 | 0.216 |
| C54:1 TAG | -0.002 | 0.022 | 0.576 | -0.003 | 0.001 | 0.105 |
| C10:2 carnitine^c^ | -0.001 | 0.024 | 0.576 | -0.001 | 0.077 | 0.426 |
| arginine | 0.004 | 0.024 | 0.576 | 0.004 | 0.015 | 0.216 |
| C6 carnitine | 0.002 | 0.025 | 0.576 | 0.003 | 0.01 | 0.216 |
| taurodeoxycholate/taurochenodeoxycholate | -0.002 | 0.026 | 0.576 | -0.002 | 0.016 | 0.216 |
| C56:2 TAG | -0.003 | 0.027 | 0.576 | -0.003 | 0.005 | 0.167 |
| C36:0 DAG | -0.004 | 0.028 | 0.576 | -0.006 | 0.002 | 0.112 |
| C30:0 DAG | -0.001 | 0.029 | 0.589 | -0.002 | 0.006 | 0.167 |
| C36:2 DAG or TAG fragment | -0.002 | 0.032 | 0.614 | -0.003 | 0.015 | 0.216 |
| C58:10 TAG | 0.001 | 0.038 | 0.622 | 0.002 | 0.013 | 0.216 |
| sphingosine | 0.002 | 0.039 | 0.622 | 0.002 | 0.015 | 0.216 |
| C36:2 DAG | -0.002 | 0.041 | 0.622 | -0.003 | 0.018 | 0.216 |
| C20:3 CE | 0.003 | 0.042 | 0.622 | 0.005 | 0.002 | 0.112 |
| Phenyllactate^c^ | -0.003 | 0.042 | 0.622 | -0.002 | 0.069 | 0.420 |
| C20:4 CE | 0.003 | 0.043 | 0.622 | 0.005 | 0.003 | 0.147 |
| C32:1 DAG | -0.002 | 0.043 | 0.622 | -0.002 | 0.016 | 0.216 |
| C5 carnitine | 0.002 | 0.043 | 0.622 | 0.002 | 0.043 | 0.312 |
| C54:2 TAG | -0.002 | 0.044 | 0.622 | -0.003 | 0.004 | 0.167 |
| C16:1 CE | 0.003 | 0.045 | 0.622 | 0.004 | 0.008 | 0.182 |
| ribothymidine | 0.002 | 0.046 | 0.622 | 0.003 | 0.018 | 0.216 |
| taurocholate | -0.002 | 0.047 | 0.622 | -0.002 | 0.017 | 0.216 |
| C3 carnitine^c^ | 0.002 | 0.05 | 0.641 | 0.002 | 0.065 | 0.403 |

^a^ Ajusting for race as White; Black; Hispanic; Other – results are as those presented in Table 2

^b^ Adjusting for the first four principal components

^c^ p>0.05

**^d^** *Computed* *according to the Benjamini Hochberg Procedure*

**Table S4**: Metabolites significantly interacting with age in BDR stratified by race in the CAMP population

| **Metabolite** | **White (n=1007 samples from 400 participants)** | | **Black (n=198 samples from 82 subjects)** | | **Hispanic (n=133 samples from 56 subjects)** | |
| --- | --- | --- | --- | --- | --- | --- |
|  | **Beta** | **Interaction p-value** | **Beta** | **Interaction p-value** | **Beta** | **Interaction p-value** |
| 2-hydroxyglutarate | -0.003 | 0.018 | -0.011 | 0.010 | -0.008 | 0.190 |
| adipate | -0.004 | 0.002 | -0.007 | 0.056 | 0.006 | 0.342 |
| GABA | 0.003 | 0.014 | 0.007 | 0.125 | 9.7x10^-5^ | 0.988 |
| 2-O-methyladenosine | 0.001 | 0.164 | 4.1x10^-4^ | 0.881 | 0.007 | 0.047 |
| 3-methyladipate/pimelate | -0.002 | 0.074 | -0.005 | 0.070 | -0.002 | 0.652 |
| C18:1 CE | 0.006 | 0.002 | 3.0x10^-4^ | 0.960 | 0.002 | 0.847 |
| ectoine | -0.001 | 0.134 | -0.003 | 0.144 | -0.004 | 0.185 |
| saccharin | 0.001 | 0.070 | 0.004 | 0.063 | -0.001 | 0.807 |
| C18:3 CE | 0.006 | 3.4x10^-4^ | -0.004 | 0.416 | 0.005 | 0.532 |
| sebacate | -0.001 | 0.204 | -0.004 | 0.111 | -0.003 | 0.470 |
| suberate | -0.002 | 0.115 | -0.006 | 0.067 | -1.4x10^-4^ | 0.981 |
| C36:1 DAG | -0.003 | 0.001 | 0.002 | 0.495 | 2.8x10^-4^ | 0.952 |
| linoleoyl ethanolamide | 0.002 | 0.007 | 0.001 | 0.527 | -0.002 | 0.718 |
| C18:0 CE | 0.003 | 0.063 | 0.008 | 0.076 | 0.001 | 0.933 |
| C22:5 CE | 0.003 | 0.018 | -0.001 | 0.858 | 0.008 | 0.100 |
| C16:0 CE | 0.006 | 0.009 | -0.001 | 0.887 | -6.2x10^-5^ | 0.994 |
| cortisone | 0.002 | 0.036 | 0.004 | 0.262 | 2.2x10^-4^ | 0.970 |
| C54:1 TAG | -0.003 | 0.005 | 0.003 | 0.335 | 0.002 | 0.625 |
| C10:2 carnitine | -0.001 | 0.109 | -0.001 | 0.709 | -0.004 | 0.105 |
| arginine | 0.004 | 0.013 | 0.006 | 0.433 | -0.004 | 0.733 |
| C6 carnitine | 0.002 | 0.043 | 0.003 | 0.248 | 0.001 | 0.884 |
| taurodeoxycholate/ taurochenodeoxycholate | -0.002 | 0.007 | 0.002 | 0.408 | -0.004 | 0.200 |
| C56:2 TAG | -0.002 | 0.062 | 0.003 | 0.480 | 0.002 | 0.755 |
| C36:0 DAG | -0.005 | 0.008 | 0.002 | 0.729 | -0.001 | 0.877 |
| C30:0 DAG | -0.001 | 0.016 | -0.003 | 0.159 | -0.001 | 0.651 |
| C36:2 DAG or TAG fragment | -0.003 | 0.029 | 0.004 | 0.267 | -0.004 | 0.486 |
| C58:10 TAG | 0.002 | 0.008 | -0.001 | 0.836 | 0.000 | 0.918 |
| sphingosine | 0.003 | 0.023 | 0.002 | 0.450 | -0.007 | 0.192 |
| C36:2 DAG | -0.003 | 0.025 | 0.003 | 0.483 | -0.001 | 0.804 |
| C20:3 CE | 0.005 | 0.004 | -0.007 | 0.210 | 0.004 | 0.539 |
| phenyllactate | -0.001 | 0.563 | -0.007 | 0.173 | -0.011 | 0.091 |
| C20:4 CE | 0.004 | 0.030 | 0.001 | 0.849 | 0.004 | 0.655 |
| C32:1 DAG | -0.002 | 0.019 | -0.004 | 0.235 | 0.001 | 0.848 |
| C5 carnitine | 0.003 | 0.038 | 0.002 | 0.698 | 0.003 | 0.629 |
| C54:2 TAG | -0.003 | 0.009 | 0.005 | 0.138 | 0.004 | 0.500 |
| C16:1 CE | 0.004 | 0.021 | -0.005 | 0.384 | 0.011 | 0.142 |
| ribothymidine | 0.003 | 0.024 | 0.005 | 0.282 | -0.008 | 0.189 |
| taurocholate | -0.003 | 0.001 | 0.004 | 0.111 | -0.002 | 0.581 |
| C3 carnitine | 0.002 | 0.069 | 0.005 | 0.244 | 0.003 | 0.690 |

*All interaction q-values computed* *according to the Benjamini Hochberg Procedure were >0.999*

**Table S5**: Sex Stratified Age*metabolite interactions for BDR outcome in the CAMP cohort

|  | **Females (n=206)^a^** | | | **Males (n=359)^b^** | | |
| --- | --- | --- | --- | --- | --- | --- |
|  | **Beta** | **Interaction**  **p-value** | ***Interaction***  ***q-value^c^*** | **Beta** | **Interaction**  **p-value** | ***Interaction***  ***q-value^c^*** |
| **2-hydroxyglutarate** | -0.004 | 0.053 | 0.124 | -0.005 | 0.002 | 0.014 |
| **C18:1 CE** | 0.005 | 0.125 | 0.219 | 0.005 | 0.031 | 0.072 |
| **C16:0 CE** | 0.003 | 0.328 | 0.328 | 0.005 | 0.046 | 0.081 |
| **GABA** | 0.002 | 0.195 | 0.228 | 0.005 | 0.006 | 0.021 |
| **C18:0 CE** | 0.007 | 0.005 | 0.035 | 0.002 | 0.232 | 0.271 |
| **C20:4 CE** | 0.003 | 0.183 | 0.228 | 0.003 | 0.147 | 0.206 |
| **ribothymidine** | 0.004 | 0.013 | 0.046 | 0.001 | 0.432 | 0.432 |

*^a^ 513 samples across 3 time-points from 206 females*

*^b^905 samples across 3 time-points from 359 males*

*GABA -* Gamma-Aminobutyric acid

CE- Cholesterol Ester

**^c^** *Computed* *according to the Benjamini Hochberg Procedure*

**Table S6:**  Sex Stratified Age*metabolite interactions for BDR outcome in the GACRS population

|  | **Females (n=135)** | | | **Males (n=185)** | | |
| --- | --- | --- | --- | --- | --- | --- |
|  | **Beta** | **Interaction**  **p-value** | ***Interaction***  ***q-value* ^a^** | **Beta** | **Interaction**  **p-value** | ***Interaction   q-value* ^a^** |
| **2-hydroxyglutarate** | -0.005 | 0.580 | *0.993* | -0.021 | 0.015 | *0.034* |
| **C18:1 CE** | -2.1x10^-4^ | 0.989 | *0.993* | 0.042 | 0.009 | *0.034* |
| **C16:0 CE** | 0.001 | 0.943 | *0.993* | 0.039 | 0.019 | *0.034* |
| **GABA** | 0.007 | 0.379 | *0.993* | 0.012 | 0.138 | *0.138* |
| **C18:0 CE** | -0.001 | 0.938 | *0.993* | 0.018 | 0.062 | *0.073* |
| **C20:4 CE** | -0.001 | 0.960 | *0.993* | 0.029 | 0.029 | *0.041* |
| **ribothymidine** | 6.6x10^-5^ | 0.993 | *0.993* | 0.021 | 0.020 | *0.034* |

*GABA -* Gamma-Aminobutyric acid

CE- Cholesterol Ester

**^a^** *Computed* *according to the Benjamini Hochberg Procedure*

**eFigures**

**Figure S1:** Correlation between 39 metabolites that interact with Age in BDR measured at baseline in CAMP

**
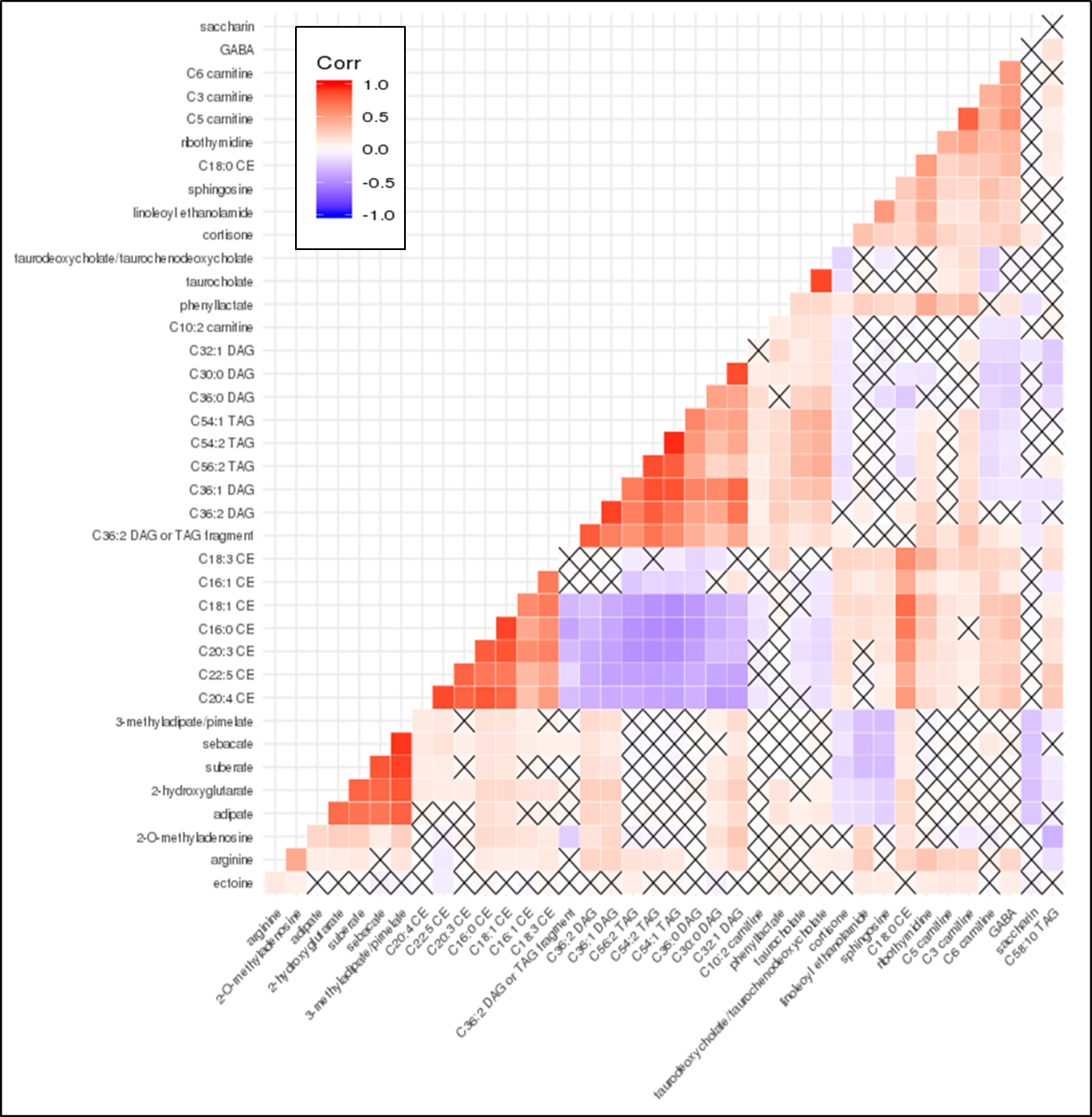
**

*Color indicates significance (the darker the color the more significant), and direction of correlation. X indicates correlation was non-significant (p<0.05)*

*GABA -* Gamma-Aminobutyric acid

CE- Cholesterol Ester

DAG- Diacylglycerol

TAG-Triacylglycerol

**Figure S2:** Correlation between 39 metabolites that interact with Age in BDR measured at study end point in CAMP

**
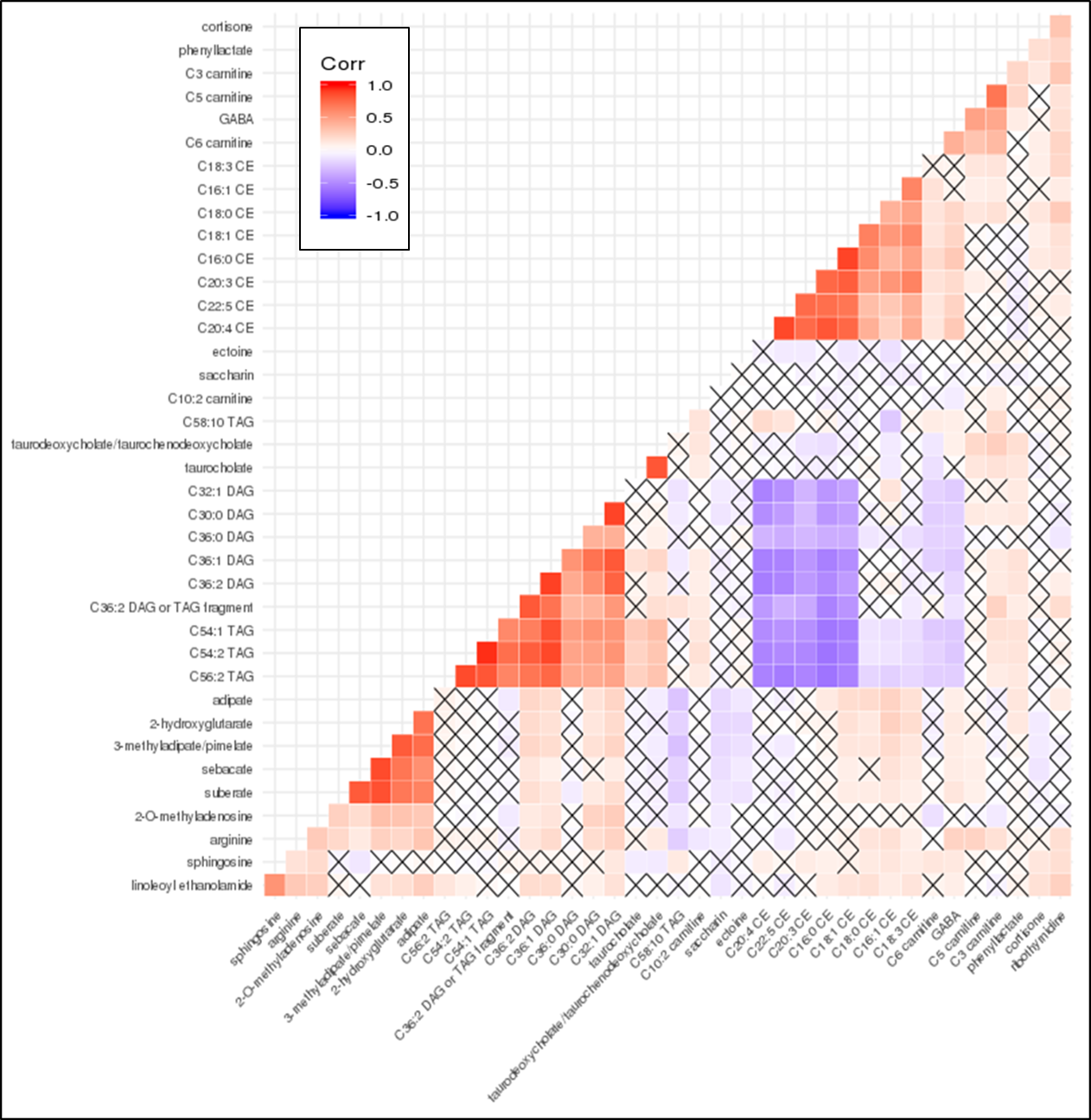
**

*Color indicates significance (the darker the color the more significant), and direction of correlation. X indicates correlation was non-significant (p<0.05)*

*GABA -* Gamma-Aminobutyric acid

CE- Cholesterol Ester

DAG- Diacylglycerol

TAG-Triacylglycerol

**Figures S3:** Correlation between 39 metabolites that interact with Age in BDR measured at study follow-up in CAMP

**
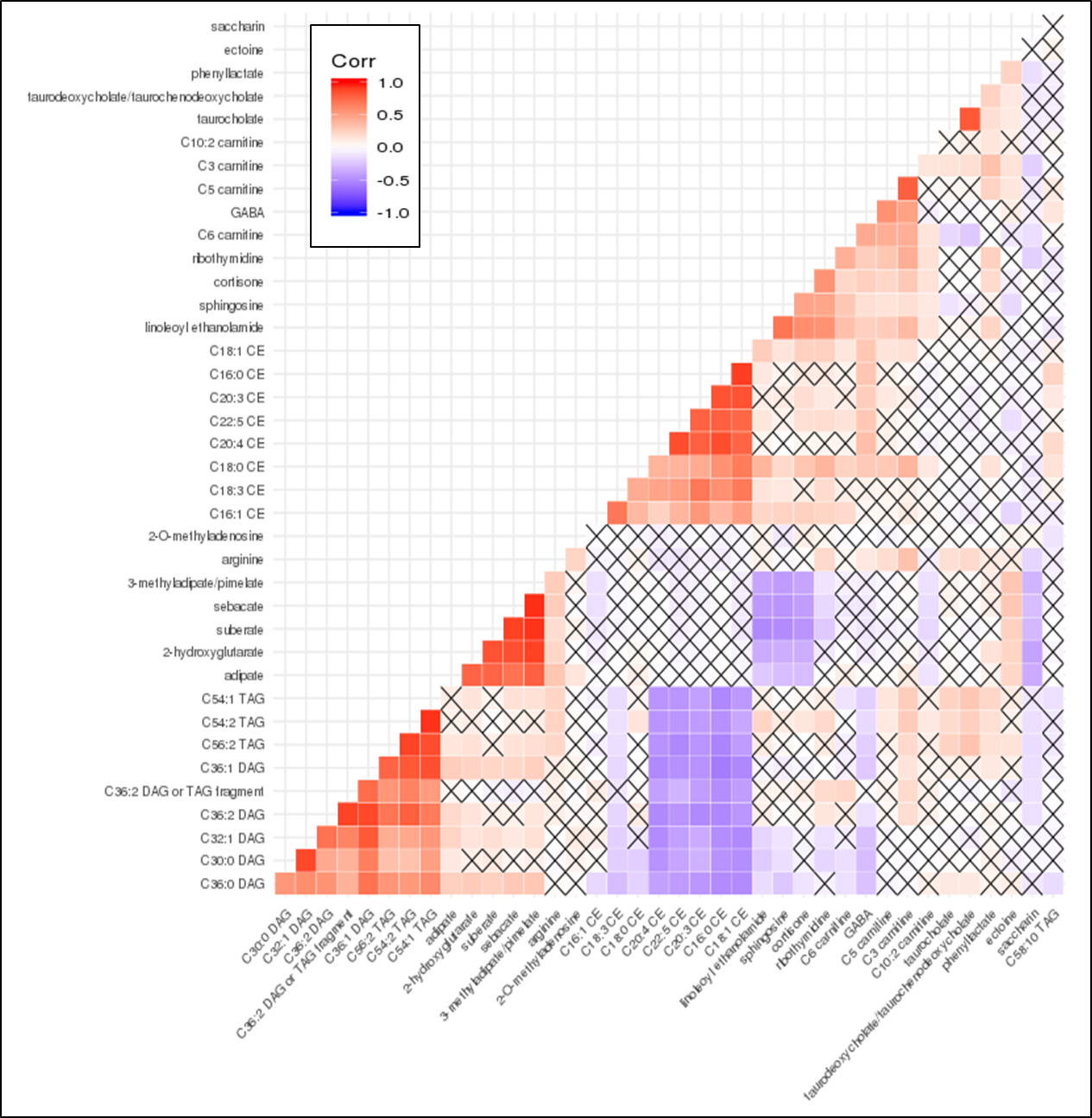
**

*Color indicates significance (the darker the color the more significant), and direction of correlation. X indicates correlation was non-significant (p<0.05)*

*GABA -* Gamma-Aminobutyric acid

CE- Cholesterol Ester

DAG- Diacylglycerol

TAG-Triacylglycerol

**Figures S4:** Relationship between Age and BDR stratified by plasma levels of 2-hydroxyglutarate in GACRS


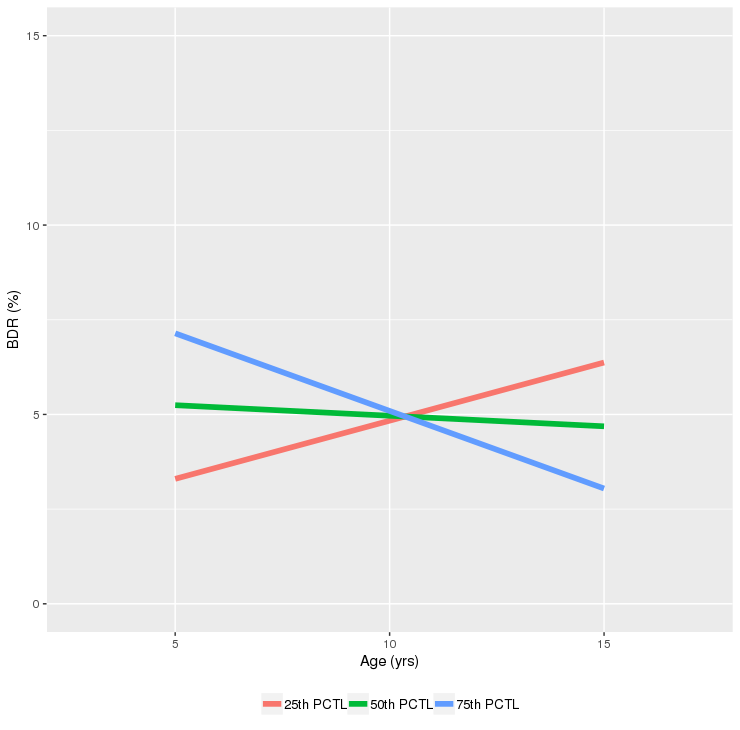


*PCTL- Percentile*

**Figures S5:** Relationship between Age and BDR stratified by plasma levels of GABA; ribothymidine C18:1 CE; C16:0 CE; C18:0 CE; C20:4 CE in GACRS


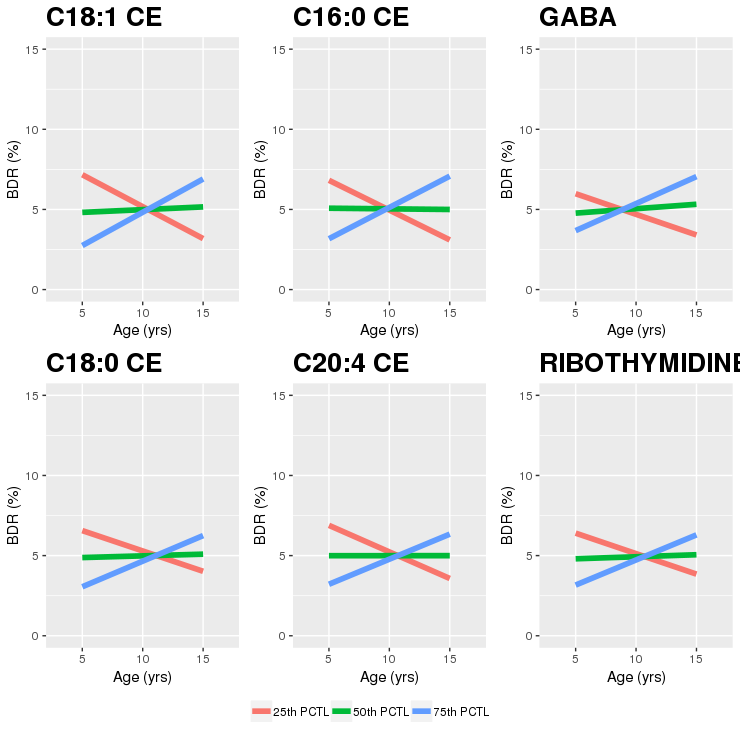


*PCTL- Percentile*

*GABA -* Gamma-Aminobutyric acid

CE- Cholesterol Ester

**Figures S6:** Relationship between Age and BDR stratified by plasma levels of 2-hydroxyglutarate in males and in females from CAMP

**
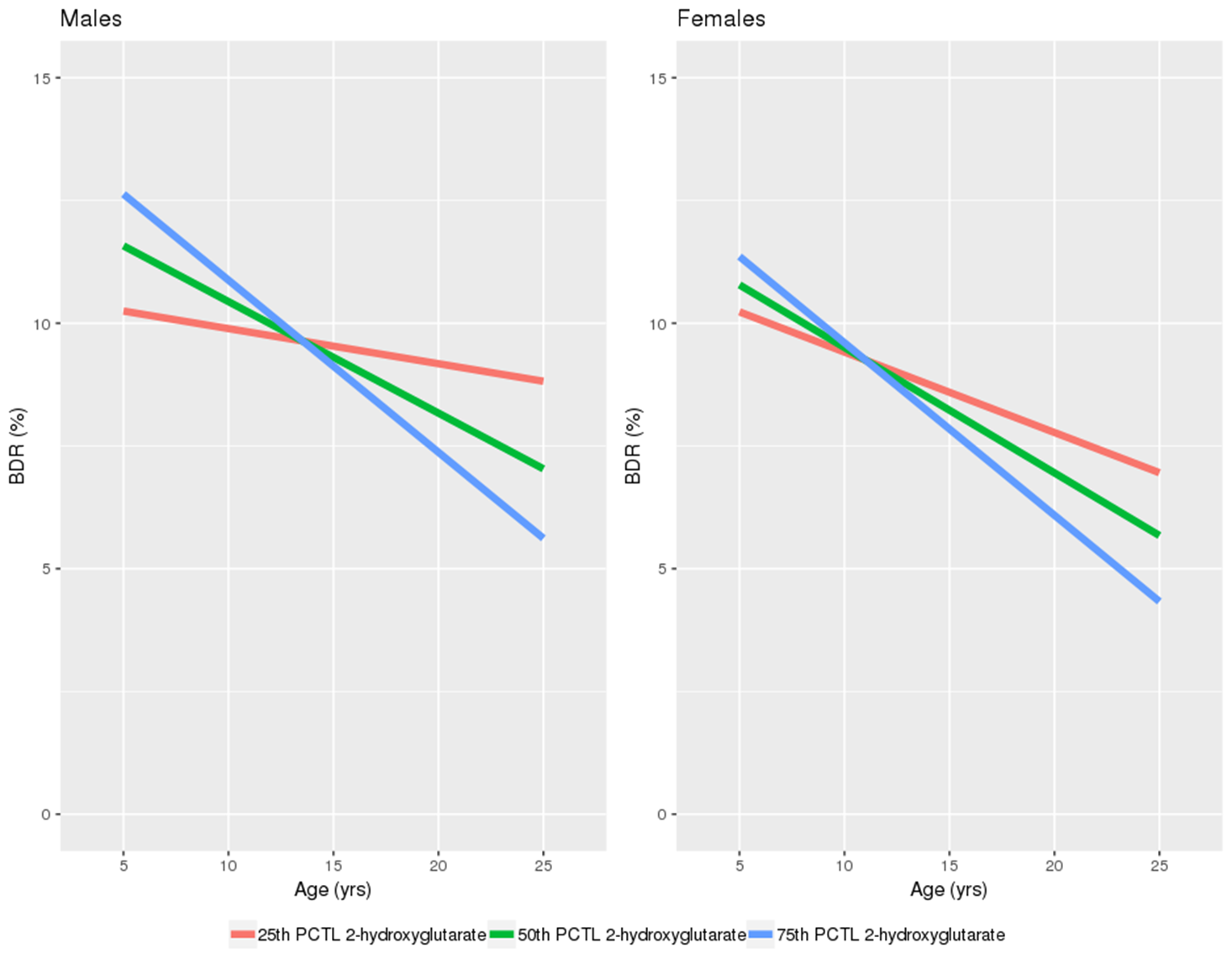
**

*PCTL- Percentile*

**Figures S7:** Relationship between age and BDR; raw data stratified by plasma levels of 2-hydroxyglutarate in CAMP (0-25th percentile (Q1), 25-75th percentile (Q2) and 75-100th percentile (Q3))

The black line is a spline fit to the data using the ggplot2 R package. The subject's trajectories are color coordinated based on the intensity of the given metabolite as seen in the legend to the right of the plot.

**Figures S8:** Relationship between age and BDR for the raw data stratified by plasma levels of C18:1 CE in CAMP (0-25th percentile (Q1), 25-75th percentile (Q2) and 75-100th percentile (Q3))

The black line is a spline fit to the data using the ggplot2 R package. The subject's trajectories are color coordinated based on the intensity of the given metabolite as seen in the legend to the right of the plot.

**Figures S9:** Relationship between age and BDR for the raw data stratified by plasma levels of C16:0 CE in CAMP (0-25th percentile (Q1), 25-75th percentile (Q2) and 75-100th percentile (Q3))

The black line is a spline fit to the data using the ggplot2 R package. The subject's trajectories are color coordinated based on the intensity of the given metabolite as seen in the legend to the right of the plot.

**Figures S10:** Relationship between age and BDR for the raw data stratified by plasma levels of C18:0 CE in CAMP (0-25th percentile (Q1), 25-75th percentile (Q2) and 75-100th percentile (Q3))

The black line is a spline fit to the data using the ggplot2 R package. The subject's trajectories are color coordinated based on the intensity of the given metabolite as seen in the legend to the right of the plot.

**Figures S11:** Relationship between age and BDR for the raw data stratified by plasma levels of C20:4 CE in CAMP (0-25th percentile (Q1), 25-75th percentile (Q2) and 75-100th percentile (Q3)).

The black line is a spline fit to the data using the ggplot2 R package. The subject's trajectories are color coordinated based on the intensity of the given metabolite as seen in the legend to the right of the plot.

**Figures S12:** Relationship between age and BDR for the raw data stratified by plasma levels of GABA in CAMP (0-25th percentile (Q1), 25-75th percentile (Q2) and 75-100th percentile (Q3)). The black line is a spline fit to the data using the ggplot2 R package. The subject's trajectories are color coordinated based on the intensity of the given metabolite as seen in the legend to the right of the plot.

**Figures S13:** Relationship between age and BDR for the raw data stratified by plasma levels of Ribothymidine in CAMP (0-25th percentile (Q1), 25-75th percentile (Q2) and 75-100th percentile (Q3)).

The black line is a spline fit to the data using the ggplot2 R package. The subject's trajectories are color coordinated based on the intensity of the given metabolite as seen in the legend to the right of the plot.

**Power analyses for the CAMP and GACRS studies:**

In order to perform an empirical power analyses of the interaction of 2 normally distributed traits on a multivariate normally distributed outcome in an unbalanced longitudinal study design, we have created an R package that is publicly available on one of the author’s GitHub site (<https://github.com/SharonLutz/ePowerLI>), which includes a tutorial detailing how the analyses are run.

For the power analyses, we based the sample sizes on the 3 visits in the CAMP study (n=560, 563, 295) and the 1 visit in the GACRS study (n=320). We used the mean and standard deviation of age for the 3 study visits in CAMP and 1 visit in GACRS to generate a normally distributed variable that represents age. We then used the mean and standard deviation of the 7 metabolites highlighted in the paper to generate a normally distributed variable that represent the given metabolites. A variable representing the outcome BDR was generated from a multivariate normal distribution with the mean generated from an interaction with the 2 normally distributed variable representing age and the metabolite with parameters based on the observed data and the variance covariance matrix based on the observed data. The random intercept model was fit for the CAMP study using the lme function in the nlme package in R and a linear regression was fit using the lm function in R for the GACRS study. The simulation studies were run for 1000 replications with the effect size of the interaction varying from the observed value to 0.02 by segments of 10. The next 2 **Figures S14-15** display the results of the power analyses for the CAMP and GACRS studies, respectively.

As seen in the **Figure S14**, the power was modest to low for all 7 metabolites, but highest for the main result, 2-hydroxyglutarate in the CAMP study. As seen in the **Figure S15**, the power was relatively higher for all 7 metabolites, especially for C18:1 CE, C16:0 CE, C20:4 CE, and 2-hydroxyglutarate in the GACRS study.

**Figures S14:** Power analysis in the CAMP study. The x-axis is the effect size of the interaction between age and the given metabolite and the y-axis is the empirical power.

**Figures S15:** Power analysis in GACRS study. The x-axis is the effect size of the interaction between age and the given metabolite and the y-axis is the empirical power.

**Figures S16:** QQ plot for the age by metabolite interaction on BDR in the CAMP study

**
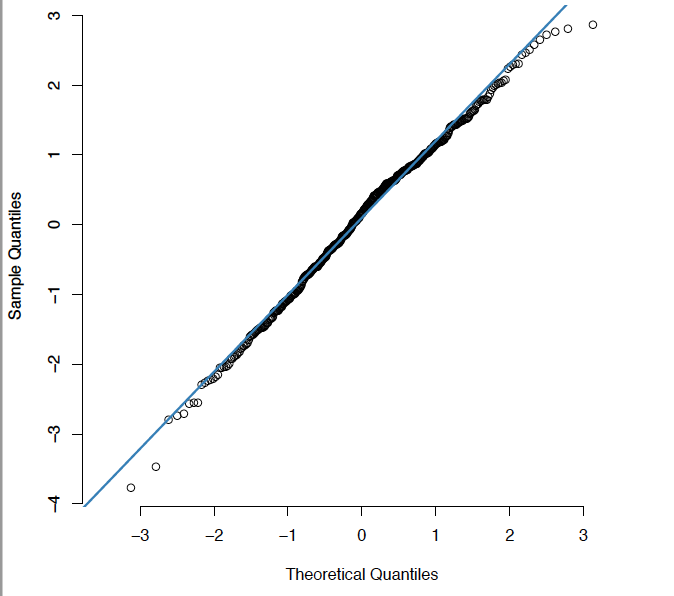
**

**References:**

1. Townsend MK, Clish CB, Kraft P, et al. Reproducibility of metabolomic profiles among men and women in 2 large cohort studies. *Clin Chem.* 2013;59(11):1657-1667.

2. Mascanfroni ID, Takenaka MC, Yeste A, et al. Metabolic control of type 1 regulatory T cell differentiation by AHR and HIF1-alpha. *Nat Med.* 2015;21(6):638-646.

3. O'Sullivan JF, Morningstar JE, Yang Q, et al. Dimethylguanidino valeric acid is a marker of liver fat and predicts diabetes. *J Clin Invest.* 2017.

4. Rowan S, Jiang S, Korem T, et al. Involvement of a gut-retina axis in protection against dietary glycemia-induced age-related macular degeneration. *Proc Natl Acad Sci U S A.* 2017;114(22):E4472-E4481.

5. Paynter NP, Balasubramanian R, Giulianini F, et al. Metabolic Predictors of Incident Coronary Heart Disease in Women. *Circulation.* 2018;137(8):841-853.
